# On the Difference in Quality between Current Heuristic and Optimal Solutions to the Protein Structure Alignment Problem

**DOI:** 10.1155/2013/459248

**Published:** 2012-12-23

**Authors:** Mauricio Arriagada, Aleksandar Poleksic

**Affiliations:** ^1^Department of Computer Science, School of Engineering, Pontificia Universidad Católica de Chile, 4860 Avenue Vicuña Mackenna, 6904411 Santiago, Chile; ^2^Department of Computer Science, University of Northern Iowa, 1227 West 27th Street, Cedar Falls, IA 50613, USA

## Abstract

The importance of pairwise protein structural comparison in biomedical research is fueling the search for algorithms capable of finding more accurate structural match of two input proteins in a timely manner. In recent years, we have witnessed rapid advances in the development of methods for approximate and optimal solutions to the protein structure matching problem. Albeit slow, these methods can be extremely useful in assessing the accuracy of more efficient, heuristic algorithms. We utilize a recently developed approximation algorithm for protein structure matching to demonstrate that a deep search of the protein superposition space leads to increased alignment accuracy with respect to many well-established measures of alignment quality. The results of our study suggest that a large and important part of the protein superposition space remains unexplored by current techniques for protein structure alignment.

## 1. Introduction

Pairwise protein structure alignment is one of the most important problems in computational molecular biology. At the same time, protein structure alignment is a very difficult problem, due to an infinite number of possible ways to position a pair of proteins in the three-dimensional space. Because of the enormous size of the search space, the research into protein structure alignment has been traditionally focused on the development of methods with better objective functions, that explore a relatively small but representative set of proteins' spatial superpositions.

In this paper, we take a different approach and study the benefits of searching proteins' superpositions in a more detailed manner. We demonstrate significant increase in the alignment accuracy of several well-known distance-based alignment methods, obtained by utilizing the superpositions that rigorously optimize a very simple and intuitive alignment metric, defined as the largest number of residues from the input proteins that can be fit under a predefined distance cutoff. 

The size of gap between the accuracy of current heuristic solutions and optimal solutions, observed in this study, suggests that the protein structure alignment problem will likely remain a hot topic in years to come.

## 2. Materials and Methods

Our study is carried out using two protein structure alignment benchmarks: *Sisyphus *and *FSSP*. In both benchmarks, an in-house algorithm, MaxPairs [1], is applied to compute the superpositions that closely approximate the measure *CA* ≤ *d*, which is defined as the largest number of pairs of residues from the input proteins that can be fit under *d* Ångströms. MaxPairs algorithm is based on the approximation algorithm EPSILON-OPTIMAL [1], which is capable of finding a superposition of the input proteins that fits at least as many pairs of residues under the distance *d* + *ε* as an optimal superposition fits under the distance *d*, for any accuracy threshold *ε* > 0. As an approximation algorithm, EPSILON-OPTIMAL suffers from high computational complexity. The algorithm's run time is a high degree polynomial in the lengths of the structures being compared. To circumvent high computational cost, the present study utilizes MaxPairs—a heuristic version of EPSILON-OPTIMAL that searches through a relatively small subset of the space of all superpositions of the input proteins inspected by EPSILON-OPTIMAL. While still not practical, as demonstrated in [1], MaxPairs enjoys accuracy superior to that of some widely utilized alignment programs and, as such, this algorithm is an indispensable tool for assessing the precision of more efficient and more popular algorithms. In present study, we set the distance cutoff to *d* = 3 Å and the accuracy threshold to *ε* = 1. Going below *ε* = 1 proves to be computationally prohibitive with our computing infrastructure.

We evaluated the performance of three well-known methods for protein structure comparison, STRUCTAL [2–4], TM-align [5], and LOCK2 [6, 7], before and after replacing their original superpositions with superpositions that optimize *CA* ≤ *d*. 

It is important to emphasize that our experiment is not designed to compare these three methods head-to-head, but rather to assess the extent of improvements in the accuracy of each method that can be made by exploring the search space in a more thorough manner. 

In choosing the methods for our study, we only considered the availability of software and the simplicity of implementing the alignment scoring functions (see the Results section). An overview of the three algorithms is given below.

**STRUCTAL**

The STRUCTAL algorithm [2–4] employs iterative dynamic programming to balance the *cRMS* score with the lengths of aligned regions. In each iteration, the algorithm computes an optimal residue-residue correspondence (alignment) of the input proteins *a* = (*a*_1_,…, *a*_*m*_) and *b* = (*b*_1_,…, *b*_*n*_) and then finds a superposition that minimizes *cRMS* of the aligned subchains (*a*_*i*_1__,…, *a*_*i*_*k*__) and (*b*_*i*_1__,…, *b*_*i*_*k*__). The *cRMS* score is given by

(1) $$\begin{array}{c} cRMS=\sqrt{\frac{1}{k}\sum_{r=1}^{k}{||a_{i_{r}}-b_{i_{r}}||}^{2}}. \end{array}$$

The alignment step in STRUCTAL is carried out using a dynamic programming routine, which implements the following recurrence formula:

(2) $$\begin{array}{c} D\left(i,j\right)=\max\{\begin{array}{c} D\left(i-1,j-1\right)+S\left(i,j\right)\\ D\left(i-1,j\right)-10\\ D\left(i,j-1\right)-10 \end{array}\\ D\left(i,0\right)=D\left(0,j\right)=0, \end{array}$$

where

(3) $$\begin{array}{c} S\left(i,j\right)=\frac{20}{1+\left({d_{i,j}}^{2}/5\right)},\;\;d_{i,j}=||a_{i}-b_{j}||. \end{array}$$

The outputs of STRUCTAL are the subchains *p* of *a* and *q* of *b*, along with the rigidly transformed protein *b*, denoted by $\hat{b}$, and a residue-residue correspondence that maximizes the STRUCTAL score

(4) $$\begin{array}{c} \sum_{i=1}^{k}\frac{20}{1+{||a_{p_{i}}-{\hat{b}}_{q_{i}}||}^{2}/5}-10G_{p,q}, \end{array}$$

where *G*_*p*,*q*_ denotes the total number of gaps in the alignment. The STRUCTAL program used in our analysis was downloaded from http://csb.stanford.edu/levitt/Structal/.

**TM-align**

TM-align is another popular protein structure alignment program, widely used in many applications, in particular for assessing the quality of protein models generated by comparative modeling or abinitio techniques. The score matrix in TM-align is protein-length specific and is defined as

(5) $$\begin{array}{c} S\left(i,j\right)=\frac{1}{1+{\left(d_{i,j}/d_{0}\right)}^{2}}, \end{array}$$

where $d_{0}=1.24\sqrt[{3}]{L-15}-1.8$, and *L* is the length of the shorter structure [5]. In contrast to linear gap penalties employed by STRUCTAL, the gap penalties in TM-align are affine and are set to 0.6 for gap-opening and 0.0 for gap-extension [5]. An improved version of the algorithm, called Fr-TM-align, has been published [8]. The TM-align software, used in this study, was downloaded from http://zhanglab.ccmb.med.umich.edu/TM-align/.

**LOCK2**

LOCK2 [6] is an improved version of the original LOCK program [7]. It incorporates secondary structure information into the alignment process. An initial superposition is obtained by comparing the vectors of secondary structure elements. An iterative procedure is then applied to minimize *RMSD* between aligned subchains of the input proteins, using the threshold distance of 3 Å for atomic superposition. Rigid body motions for *RMSD* minimization are realized using quaternion transformations [9, 10].

The alignment returned by LOCK2 is a sequence of pairs of points

(6) $$\begin{array}{c} \left(a_{i_{1}},b_{i_{1}}\right),\ldots ,\left(a_{i_{k}},b_{i_{k}}\right),\;i_{1}<\cdots <i_{k}, \end{array}$$

where *a*_*i*_*r*__ are *b*_*i*_*r*__ each other's nearest neighbors. More specifically, for every *r* ∈ {1,…, *k*}, the point *b*_*i*_*r*__ is the closest point in protein *b* to the point *a*_*i*_*r*__ and vice versa. The final alignment is generated through a two-step process. First, for every atom *a*_*i*_ from protein *a*, the algorithm finds the nearest atom from protein *b* that is at distance ≤3 Å from *a*_*i*_. In the second step, the algorithm selects the maximum number of aligned pairs in sequential order, by removing pairs that violate colinearity.

The LOCK2 software can be downloaded from http://lock2.stanford.edu.

## 3. Results

### 3.1. Sisyphus Benchmark

The *Sisyphus *test [11] is frequently used to assess the accuracy of automated methods for protein structure comparison [1, 12]. This sophisticated benchmark utilizes 125 alignments of structurally related proteins, created by experts in the field of protein structure analysis. The reference alignments can be downloaded from http://sisyphus.mrc-cpe.cam.ac.uk. 

**Figure 1:**
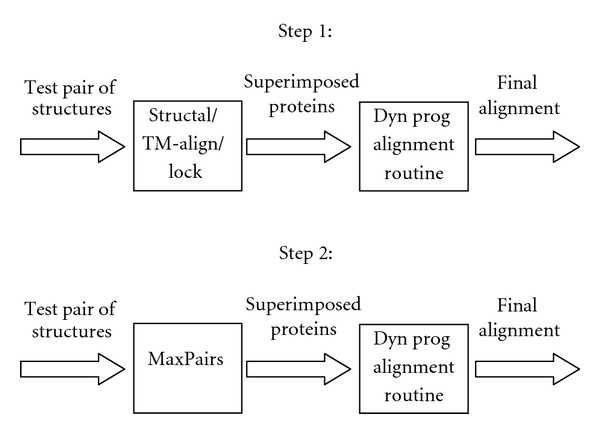
The procedure for creating methods' specific alignments and alignments based on MaxPairs superpositions.

In present study, we (like Rocha et al. [12]) utilize only a subset of the *Sisyphus* test set, containing 106 alignments between single-chain proteins. The two-step process is illustrated in Figure 1. In the first step, STRUCTAL, TM-align, and LOCK2 are run with default parameters to generate the methods' specific alignments between proteins from the *Sisyphus *set. These alignments are then compared to the reference (“gold-standard”) alignments to compute the percentage of correctly aligned residue pairs [1, 12].

In the second step, the MaxPairs algorithm is run to compute the set of (near-)optimal superpositions, namely, the superpositions that rigorously maximize the number of pairs of atoms that can be fit under 3 Å. We used our own implementations of the STRUCTAL, TM-align, and LOCK2 alignment procedures to compute optimal residue-residue correspondence (alignment) between the newly superimposed proteins. The percentage agreement with reference alignments is recorded again and compared to the agreement obtained in the first step.

The agreement with reference alignments in the *Sisyphus* test is defined as a function of the magnitude of the alignment error. More specifically, for the alignment tolerance shift *s*, the agreement is defined as *I*_*s*_/*L*_ref_, where *I*_*s*_ is the number of aligned residues that are shifted by no more than *s* positions in the reference alignment and *L*_ref_ is the length of the reference alignment [12]. The perfect agreement is the one that corresponds to zero-shift (*s* = 0).

**Figure 2:**
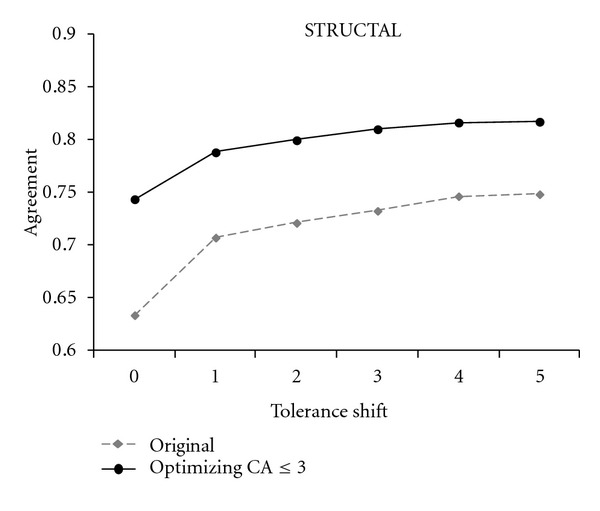
The accuracy of the STRUCTAL algorithm using original versus optimized superpositions in the *Sisyphus* benchmark.

**Figure 3:**
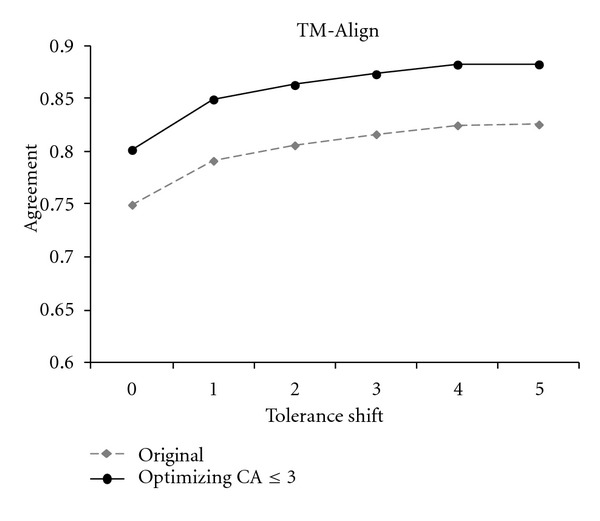
The agreement of TM-align alignments and reference alignments in the *Sisyphus* benchmark.

**Figure 4:**
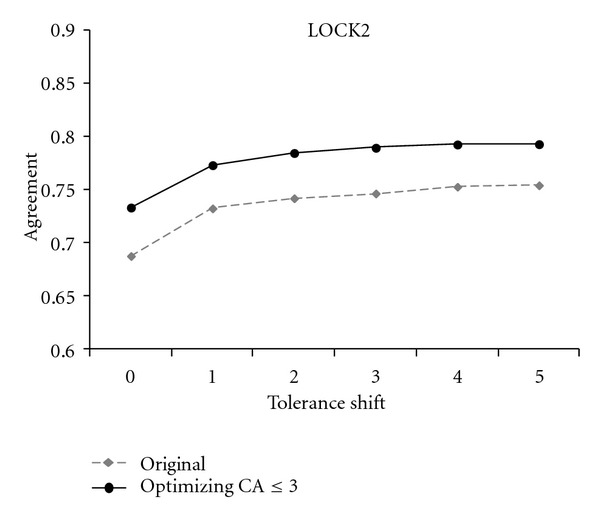
The agreement of LOCK2 alignments and reference alignments in the *Sisyphus* benchmark.

The dashed lines in Figures 2, 3, and 4 track the performance of original STRUCTAL, TM-align, and LOCK2 methods. The solid lines show the performance of the same methods when run on the superpositions that maximize the number of residues under 3 Å. As seen in these figures, there is a significant boost in the methods' accuracy resulting from the “fine-tooth comb” search of superposition space. More precisely, the new superpositions improve absolute agreement with the reference alignments for STRUCTAL, TM-align, and LOCK2 by 11%, 5%, and 5%, respectively, with a similar trend continuing for nonzero shift.

**Figure 5:**
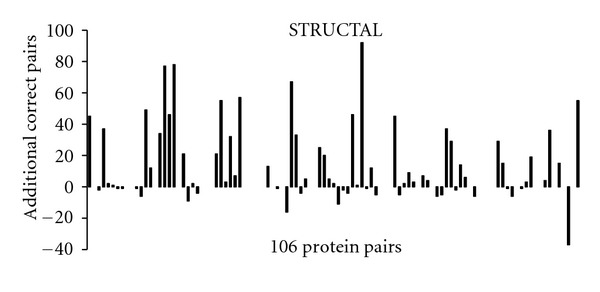
The increase in accuracy of STRUCTAL obtained on 106 pairs from the *Sisyphus* benchmark.

**Figure 6:**
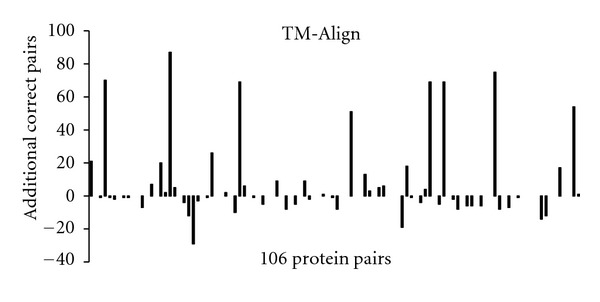
The increase in accuracy of TM-align obtained on 106 pairs from the *Sisyphus* benchmark.

**Figure 7:**
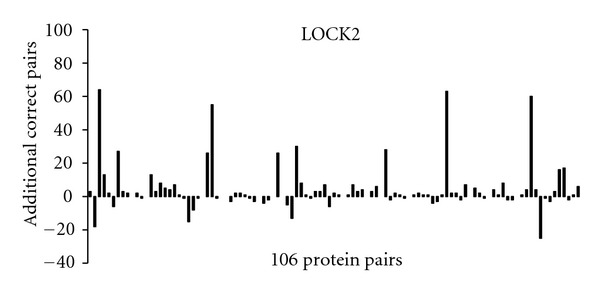
The increase in accuracy of LOCK2 obtained on 106 pairs from the *Sisyphus* benchmark.

The increase in number of correctly aligned residues, obtained by switching to MaxPairs superpositions, varies from one pair of structures to another (Figures 5, 6, and 7). For some pairs, the difference is striking. However, it should be emphasized that, in some of these cases, such a high difference might be due to unavailability of information in PDB files used by the methods in our study. For instance, the LOCK method is built to take advantage of the residues' secondary structure assignment. Hence, it is reasonable to assume that the lack of secondary structure information in the PDB file for one or both structures will often decrease the accuracy of the LOCK alignment of those structures.

A more detailed analysis shows that, when MaxPairs superpositions are used, the number of residue pairs correctly aligned by STRUCTAL increases by more than 10 for 31 out of 106 test pairs. The corresponding number of test pairs for which the same magnitude of increase is observed for TM-align and LOCK is 14 and 13, respectively. For comparison, original STRUCTAL superpositions have such an advantage only in 3 out of 106 test pairs. For TM-align and LOCK, the corresponding numbers are 5 and 4.

The value added by the deep search of superposition space makes some of the methods analyzed here comparable to the best to date methods evaluated in the *Sisyphus* test. A slight accuracy advantage of algorithms such as Matt [13], PPM [14], and ProtDeform [12] is due to the fact that these methods consider proteins as flexible, rather than rigid objects. In other words, unlike STRUCTAL, TM-align, and LOCK2, which all utilize single transformations of input proteins to compute final alignments, the new generation of protein structure alignment methods consider sequences of different rigid transformations at different sites. It should be emphasized that the methods based on sequences of local transformations can themselves benefit from incorporating the “fine-tooth comb” search to detect fragments of local similarity. This would lead to further improvements in their overall accuracy, but the true extent of these improvements can only be accessed through a carefully designed study.

### 3.2. FSSP Benchmark

Our second benchmarking set utilizes 183 representative pairs of proteins, related at various levels according to FSSP structural classification [15]. This test set consists of 55 family pairs, 68 superfamily pairs, and 60-fold pairs (see Supplementary Material available online at doi:10.1155/2012/459248).

In contrast to *Sisyphus* benchmark, which compares alignments returned by automated methods to those generated by human experts, the alignment precision in the FSSP benchmark is assessed using a set of well-known alignment quality measures:

- NumPairs(d) represents the number of aligned pairs of residues in two proteins that are at distance ≤*d* Ångströms from each other. We note that, unlike *CA* ≤ *d*, which is a globally optimal metric, representing the maximum number of pairs of residues in the superimposed structures that can be placed under *d* Ångströms, NumPairs(d) represents the method specific count of pairs of aligned residues at distance ≤*d*.
- *Similarity Index*, denoted by SI, is defined as *cRMS* × min⁡⁡{*L*(*a*), *L*(*b*)}/*Nmat*, where *Nmat* is the number of aligned residues in proteins *a* and *b* and *L*(*a*) and *L*(*b*) are the lengths of *a* and *b*, respectively [16]. The *cRMS* score, used in the formula for SI, is computed based upon the method specific alignments.
- *The Percentage of Structural Similarity*, PSI(d), is defined as NumPairs(*d*)/min⁡{*L*(*a*), *L*(*b*)} (see, for example, [8]).

As seen in Table 1, a more detailed search of the superposition space increases both NumPairs and PSI scores for all three methods in our study. The increase in SI scores is also seen for both STRUCTAL and LOCK2. It is interesting to note, though, that the original TM-align superpositions yeald better SI scores than the optimal superpositions.

The FSSP level-specific results of our benchmarking analysis are summarized in Tables 2, 3, and 4.

**Figure 8:**
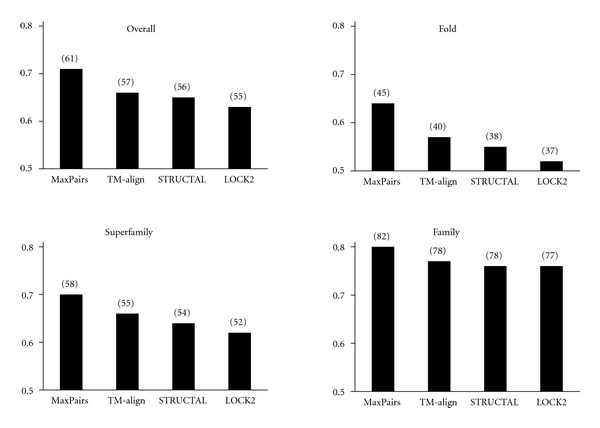
Alignment independent PSI scores in the FSSP benchmark. The number in parentheses is the highest number of pairs of residues (averaged over each test set) that can be placed under 3 Å, given the superpositions generated by each method.

Figure 8 shows the alignment independent PSI scores computed from superpositions generated by STRUCTAL, TM-align, and LOCK2. For reference, a near-optimal PSI score, averaged across the FSSP test set and computed by the MaxPairs algorithm, is also provided in this figure.

The data used in Figure 8 shows that (on average) STRUCTAL, TM-align, and LOCK fail to place 8%, 7%, and 11% pairs of residues at distance ≤3 Å, respectively. As expected, the best performance of these methods is observed at the FSSP family level (STRUCTAL fails to place 5%, TM-align: 5%, LOCK: 6%) and worst at FSSP fold level (STRUCTAL: 15%, TM-align: 12%, LOCK: 17%).

### 3.3. Illustrative Examples

**Figure 9:**
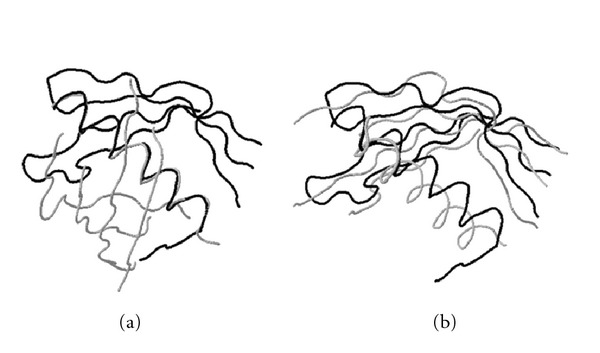
Structural alignment of two cystatin-like folds: *delta-5-3-ketosteroid isomerase from pseudomonas putida*, PDB ID: 1opy (black) and *chicken egg white cystatin*, PDB ID: 1cewI (gray), obtained by (a) a heuristic method and (b) MaxPairs. For simplicity of presentation, we hide the parts of two structures that are not well superimposed by either program. In this particular test case, switching to MaxPairs superpositions yields a twofold increase in the number of pairs of residues that can be fit under 3 Å (59 versus 29). The corresponding percentage increase for the distance cutoff of 5 Å is 76% (65 versus 37 pairs).

**Figure 10:**
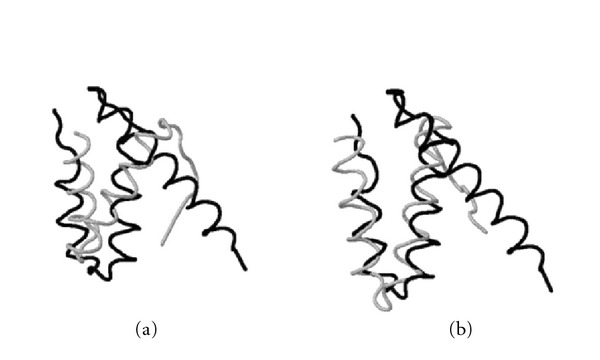
Structural superposition of the *urease from Bacillus pasteurii*, PDB ID: 1ubpA (black) and the* dynein light chain 1 from human*, PDB ID: 1cmiA (gray), obtained by (a) a heuristic method and (b) MaxPairs (we hide misaligned C-terminal regions from both structures). In this test case, a subtle change in structural superposition, made by MaxPairs, increases the number of pairs of residues that can be fit under 3 Å from 9 to 36 (and from 16 to 42 when the cutoff distance of 5 Å is used).

**Figure 11:**
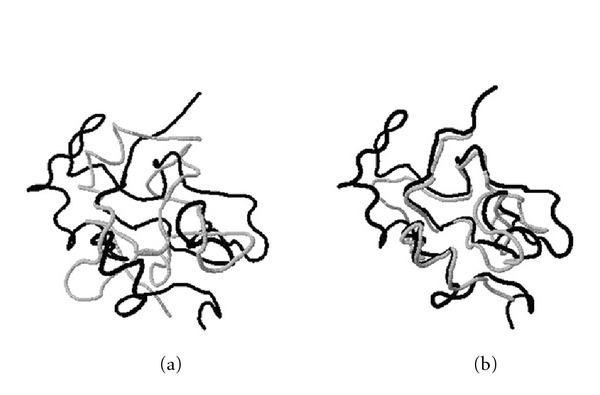
Structural superposition of *HiPIP* (high-potential iron protein) from Chromatium* vinosum*, PDB ID: 1ckuA (black) and HiPIP isolated from the phototrophic bacterium Rhodocyclus* tenuis*, PDB ID: 1isuA (gray), obtained by (a) a heuristic method and (b) MaxPairs. This is another example illustrating an obvious difference in quality of two structural matches.

**Figure 12:**
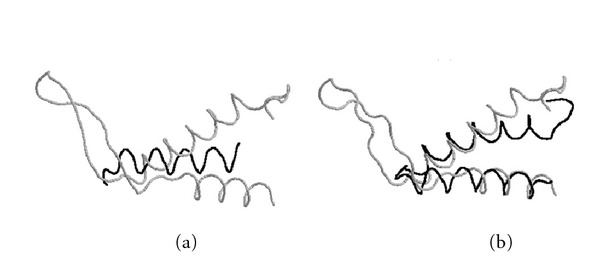
Structural superposition of two helical regions in the *elongation factor TS*, PDB ID: 1tfeA (black) and the* ribosomal protein S7*, PDB ID: 1rssA (gray), obtained by (a) a heuristic method and (b) MaxPairs. Regions not aligned well by the two programs are hidden for simplicity of presentation. When run on superpositions generated by MaxPairs, the same heuristic method aligns 22 more residues under the distance 3 Å (29 versus 7).

**Figure 13:**
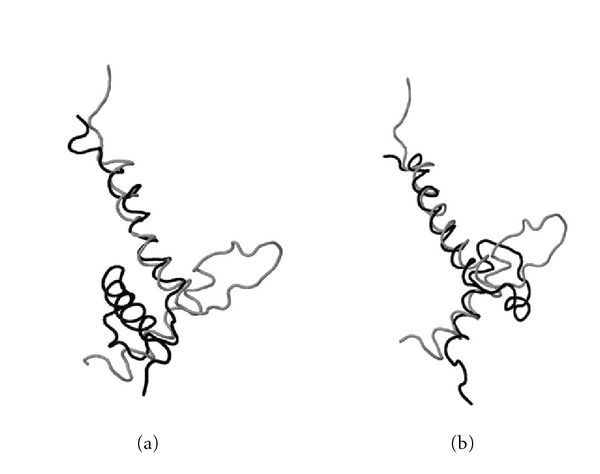
Structural superposition of the *DNA-binding domain of PHO4*, PDB ID: 1a0aA (black) and the *basic/helix-loop-helix/leucine *zipper domain of the upstream stimulatory factor, PDB ID: 1an4A (gray), obtained by (a) a heuristic method and (b) MaxPairs. Unlike the heuristic method, MaxPair is capable of aligning both helical regions from these two proteins.

Several examples illustrating the advantage of the deep search of superposition space are given in Figures 9, 10, 11, 12, and 13. 

While examples in Figures 9–13 are striking, it should be noted that they represent rather isolated cases. In fact (as the reader can conclude from Figures 5, 6, and 7), there are several examples where the output of heuristic methods compares favorably to that of MaxPairs (although the difference in quality is not as obvious as that shown in Figures 9–13). As emphasized before, in many instances, the inaccuracy of the alignment generated by heuristic methods is due to insufficient structural information stored in the PDB file, relied upon these methods.

## 4. Discussion

Resent years have witnessed advances in the development of methods for approximate and exact solution to protein structure alignment problem. One of the first such methods is the Umeyama's algorithm for finding the transformation that gives the least mean squared error between two point patterns [17]. Since then, several algorithms have been published for finding a near-optimal solution to the structure alignment problem under distance constraints. The procedure by Akutsu, for example, returns a superposition of the input proteins that fits at least as many pairs of residues under the distance *c* · *d* as an optimal alignment fits under the distance *d*, for every fixed *c* > 1 [18]. This algorithm runs on the order of *O*(*n* ^8^), where *n* denotes the protein length. An improved running time procedure for the same problem has also been published [19]. The EPSILON-OPTIMAL algorithm, used in present study, is able to place at least as many pairs of residues under the distance *d* + *ε* as an optimal superposition places under the distance *d*. The asymptotic cost of EPSILON-OPTIMAL is *O*(*n* ^4^) for globular and *O*(*n* ^8^) for nonglobular proteins [1].

The polynomial time approximation schemes (PTASs) have been designed for selected nonsequential protein structure alignment measures [20] as well as for the class of measures satisfying the so-called Lipschitz condition [21]. Moreover, methods exist that rigorously minimize proteins' intra-atomic distances, including the algorithm by Caprara et al., which is capable of approximating the “Contact Map Overlap” (CMO) measure with great accuracy [22]. Finally, the algorithms for absolute optimum, with respect to selected alignment metrics, have also been published [1, 23], but they are computationally too expensive for everyday use. 

Although inefficient for large scale analysis, the algorithms for exact solution are indispensable tools for assessing the accuracy of more commonly used heuristic methods. The present study utilizes a set of precomputed superpositions to evaluate the improvements in accuracy of three well-known protein structure alignment algorithms, obtained by the deep search of the superposition space. In the *Sisyphus* benchmark, these superpositions increase the accuracy of alignments generated by STRUCTAL, TM-align, and LOCK2 by 11%, 7%, and 6%, respectively. An improvement of similar magnitude is seen after allowing for alignment errors (residue shifts). In the FSSP benchmark, the new superpositions increase NumPairs and PSI scores for STRUCTAL, TM-align, and LOCK2 by ~7%, ~5%, and ~13%, respectively. A particularly noticeable improvement is seen in the *Similarity Index* scores of alignments generated by LOCK2 (from 8.35 to 5.69). 

We emphasize that our analysis provides an estimate of the lower bound on the difference between optimal and heuristic solution, since alignments generated by MaxPairs are not always optimal (in the strict sense).

Finally, it is reasonable to expect that a more thorough exploration of the superposition space, coupled with the fragment-based alignment techniques, can be used to further improve the precision of methods based on sequences of local transformations, such as Matt [13], PPM [14], and ProtDeform [12].

## 5. Conclusions

A typical distance-based protein structure alignment method explores the space of proteins' spatial superpositions, computing an optimal residue-residue correspondence (alignment) each time a new superposition is generated. Because of the large search space, current methods for protein structure alignment must trade precision for speed and explore only a small but representative set of superpositions. 

We utilize an algorithm capable of finding an alignment of any specified accuracy to demonstrate significant increase in the alignment quality of solutions generated by three popular protein structure alignment methods, obtained through the deep search of the superposition space. The large lower bound on the size of gap between optimal and heuristic solutions, observed in this study, suggests that the protein structure alignment problem will likely remain an attractive research area throughout the next decade. 

## Supplementary Material

FSSP benchmark consisting of as set of 183 representative pairs of proteins extracted from the FSSP database (ftp://ftp.ebi.ac.uk/pub/databases/fssp/).

## Figures and Tables

**Table 1:** Average (per-pair) accuracy of STRUCTAL, TM-align, and LOCK2 in the FSSP benchmark, for all structural levels combined. The best results are indicated in bold.

	NumPairs(3)	PSI(3)	SI
STRUCTAL			
Original	50.47	0.59	7.85
Near-optimal	**53.81**	**0.63**	**7.37**
TM-align			
Original	53.35	0.62	**5.86**
Near-optimal	**55.85**	**0.65**	5.95
LOCK2			
Original	51.75	0.60	8.35
Near-optimal	**58.46**	**0.68**	**5.69**

**Table 2:** Average accuracy of the three methods in our study, computed on 60 pairs of proteins that share the same FSSP fold.

	NumPairs(3)	PSI(3)	SI
STRUCTAL			
Original	31.48	0.47	10.07
Near-optimal	36.68	0.54	9.63
TM-align			
Original	35.98	0.52	7.60
Near-optimal	39.58	0.57	7.76
LOCK2			
Original	34.82	0.50	12.56
Near-optimal	42.47	0.61	7.25

**Table 3:** Average accuracy computed on the set of 68 pairs of proteins that belong to the same FSSP superfamily.

	NumPairs(3)	PSI(3)	SI
STRUCTAL			
Original	47.71	0.58	8.41
Near-optimal	50.09	0.61	7.61
TM-align			
Original	51.40	0.62	6.11
Near-optimal	52.71	0.64	6.10
LOCK2			
Original	48.63	0.59	8.17
Near-optimal	55.37	0.67	5.85

**Table 4:** Average accuracy computed on the set of 55 pairs of structures from the same FSSP family.

	NumPairs(3)	PSI(3)	SI
STRUCTAL			
Original	74.6	0.74	4.76
Near-optimal	77.11	0.76	4.62
TM-align			
Original	74.71	0.73	3.65
Near-optimal	77.49	0.76	3.79
LOCK2			
Original	74.09	0.73	3.98
Near-optimal	79.73	0.78	3.80
