# Supplementary material for: On the Difference in Quality between Current Heuristic and Optimal Solutions to the Protein Structure Alignment Problem
Source: Biomed Res Int. 2012 Dec 23;2013:459248. doi: 10.1155/2013/459248 (PMC3591119; doi:10.1155/2013/459248)
Supplement: Supplementary file 1 — FSSP benchmark consisting of as set of 183 representative pairs of proteins extracted from the FSSP database (ftp://ftp.ebi.ac.uk/pub/databases/fssp/). [file 459248.f1.pdf]

Structure pair	Length 1	Length 2
**Family**		
1bdo_1ghj	80	79
1d3bA_1d3bB	72	81
1aly_1c28A	146	125
1dt4A_1vih	73	71
1agdB_1cqkA	99	101
1ayoA_1aohA	130	143
1dynA_1evhA	113	111
1b20A_1rgeA	109	96
1smtA_1qbjA	98	65
2vhbA_1ash	137	147
1rlw_1bdyA	126	123
2hddA_1akhA	55	49
1lfdA_1vcbA	87	98
1r69_1b0nA	63	103
1stmA_1a34A	141	147
1aba_1kte	87	105
1dz3A_1dcfA	123	133
1d4tA_1qadA	104	104
1onc_7rsa	103	124
2irfG_1awcA	109	110
1ghqB_1cklB	129	126
1jrhI_1ten	95	89
1vpfA_1pdgA	94	87
1fit_1kpf	124	111
1lis_1gakA	131	137
1b6e_1ixxB	121	123
1kwaA_1pdr	88	96
1eteA_1hmcB	134	143
2u2fA_1h6kZ	85	78
1ifqA_1h8mA	124	140
2bopA_1f9fA	85	76
256bA_2ccyA	106	127
1tiiD_1bcpD	98	110
1rie_1rfs	127	127
2dhqA_1di0A	136	148
1cy5A_3ygsP	92	97
1fxkA_1fxkC	103	133
1jfiB_1aoiB	135	83
1ax8_1cnt3	130	146
1ibzA_2cuaA	111	122
1jb0C_1xer	80	102
1quqB_1quqA	114	121
1gcpA_1bbzA	65	58
1bh9B_1aoiA	89	98
1vls_1nfn	146	132
1aqzA_9rnt	142	104
1b9lA_1dhn	119	121
1f5wA_1tlk	124	103
3inkC_1rcb	121	129

1otgA_1dptA	125	117
1qgvA_1erv	130	105
1dwnA_1msc	127	129
1shsA_1ejfA	115	110
1vcbB_1buoA	88	121
1opy_1jn5A	123	136

Superfamily

1ag4_1f53A	103	84
1bdo_1htp	80	131
1a75A_1psrA	106	100
1stmA_1cwpA	141	149
1wdcA_1sfcC	64	77
1mjhA_1cozA	143	126
1an2A_1am9A	86	80
1b0nA_1lmb3	103	87
1qgvA_1ewxA	130	144
4hb1_1a32	44	85
1e2aA_1fzcA	102	74
1b20A_9rnt	109	104
1he1A_1nfn	135	132
1hcnB_1agqA	110	91
1x11A_1evhA	126	111
1d66A_1zmeC	57	70
1t1dA_1buoA	100	121
1akhA_1tc3C	49	51
1ytfC_1ytfD	46	100
1bkb_1mjc	132	69
1g3fA_1c9qA	117	117
1exg_1ayoA	110	130
2chsA_1qu9A	114	126
1pi2_1c2aA	61	120
1dulA_1qb2A	61	106
1b3aA_1a15A	67	67
1ptf_1ctf	87	68
1dptA_1otfA	117	59
1mof_1fzgD	53	54
1jc7A_1krs	129	110
1aoiA_1a7w	98	68
1awcA_2hts	110	88
2pkaA_2hrvA	80	139
1c28A_1sfp	125	111
1tgsI_1tbrR	56	103
1ptq_1far	50	52
1dupA_1tul	136	102
1cklB_1hfh	126	120
1jhgA_2ezk	101	93
1f3uA_1f3uB	118	139
1pfsA_1gvp	78	87
1tocR_1bpi	120	58
1bylA_1jc4A	122	139

2drpA_1aayA	63	85
1tpg_1fbr	91	93
1cof_1svy	135	101
1emn_1dx5I	82	118
1chc_1rmd	68	116
1kum_1vcbC	108	142
1bbzA_1tud	58	60
1jb0C_1a6I	80	106
1vsrA_1gefA	134	120
3caoA_3cyr	102	107
1hulA_1d9cA	108	121
1hyp_1bea	75	116
1cdzA_1imoA	96	88
1jpyB_1bndA	121	109
1jrhI_1do6A	95	124
1lea_1smtA	72	98
1a8o_1ak4C	66	145
1ekrA_1cc8A	143	72
1eo6A_1vcbA	116	98
1fseA_1fc3A	67	119
1cy5A_1ddf	92	127
2dhqA_1be1	136	137
1bazA_2cpgA	49	43
1dhn_1b66A	121	138
1hryA_1cktA	73	71

Fold

1rss_1tfe	140	142
1kp6A_1jidA	79	114
2ezh_1fseA	65	67
1awd_2igd	94	61
1qj8A_1dwnA	148	127
1g8eA_1jb0F	98	141
1bgf_1ytfB	124	46
1ctf_1vih	68	71
1rzl_1bea	91	116
1ahsA_1sfp	126	111
1i0uA_1dx5I	82	118
1diiC_1dw0A	73	112
1skz_1ejaB	104	53
1cei_1g8qA	85	90
1utg_1qc7A	70	101
1b33N_1jb0D	67	138
1rhoA_1jrhI	142	95
1i8nA_5hpgA	89	84
1pi2_1bi6H	61	41
1a02F_1g2cB	53	40
1cll_1j7rA	144	77
1ecmA_2vhbA	91	137
1ubpB_1c5eA	122	95
1hcrA_1akhA	52	49

1f08A_8atcB	148	146
1zeiA_1bg8A	53	76
1lywA_1b5fB	95	87
1bpi_1kigI	58	60
2gcc_2bb8	63	71
1i2tA_1guxB	61	141
1seiA_1tig	130	88
1esxA_1jb0J	96	41
1fc2C_2gp8A	43	40
2a3dA_1dvkA	73	149
1smpI_1hmt	100	131
1azpA_1a15A	66	67
1ag4_1wapA	103	68
1isuA_1ckuA	62	85
1cmbA_1bazA	104	49
1a1x_1dtvA	106	67
1hymA_2sniI	44	64
1f37A_1aba	109	87
1tuc_1ihvA	61	52
1jpyB_2tgi	121	112
1ucyE_2hrvA	109	139
1an4A_1a0aA	65	63
1ifqA_1pne	124	139
1jy2O_1jy2N	51	43
1r1bA_1hp8	56	68
1opy_1cewI	123	108
1g6uA_1be3H	48	64
1d3bA_1ytfC	72	46
2alcA_1d66A	65	57
1eduA_1hxiA	143	108
2occK_1ltaC	49	45
2occJ_2occL	58	47
1ubpA_1cmiA	99	85
1pru_1uxc	56	50
1poc_1poa	134	118
1b93A_1pauA	148	140
